# A Longitudinal Reservoir Model of Stress Dissipation and the Influences of Concomitant Perceptions of Control

**DOI:** 10.1093/geroni/igaa057.2054

**Published:** 2020-12-16

**Authors:** C S Bergeman, Raquael Joiner, Niccole Nelson, Pascal Deboeck

**Affiliations:** 1 University of Notre Dame, Notre Dame, Indiana, United States; 2 University of Notre Dame, Mishawaka, Indiana, United States; 3 University of Utah, Salt Lake city, Utah, United States

## Abstract

To characterize the stress regulation system, we use a reservoir to reflect how much stress an individual “holds” over time. Factors affecting what is contained in a stress reservoir are incoming stress (Input), accumulation/dissipation (Strdiss), and actions taken to discharge stress (e.g., Control). At the within person level, time-varying control predicts better Strdiss (β= -0.03±0.01, p <.001), even when controlling for between person differences (e.g., age, neuroticism) and between and within person impacts of Input. Thus, control reflects an important stress dissipation tool. Further analyses indicated a significant 2-way interaction between time-varying effects of Input and Control (β= 0.14±0.03, p <.0001) and Strdiss and Control (β= 0.60±0.18, p <.001) on self-reported health and a significant 3-way time-varying interaction of Input, Strdiss and Control on depression (β= -0.173±0.07, p <.012). Studies of this type move beyond the static assessments of risk and resilience to a more dynamic one.

